# The Use and Understanding of Mild Cognitive Impairment in Routine Specialist Care: A Survey Among German Memory Clinics

**DOI:** 10.3390/geriatrics10010021

**Published:** 2025-02-02

**Authors:** Gloria S. Benson, Claudia Bartels, Feride Stamatis, Michael Belz, Hermann Esselmann, Lutz Frölich, Lucrezia Hausner

**Affiliations:** 1 Department of Geriatric Psychiatry, Central Institute of Mental Health, Medical Faculty Mannheim, Heidelberg University, J5, 68159 Mannheim, Germany; gloria.spielmann-benson@zi-mannheim.de (G.S.B.); feride.stamatis@zi-mannheim.de (F.S.); lutz.froelich@zi-mannheim.de (L.F.); 2 Department of Psychiatry and Psychotherapy, University Medical Center Göttingen, Von-Siebold-Str. 5, 37075 Göttingen, Germany; claudia.bartels@med.uni-goettingen.de (C.B.); michael.belz@med.uni-goettingen.de (M.B.); hermann.esselmann@med.uni-goettingen.de (H.E.)

**Keywords:** mild cognitive impairment, mild cognitive impairment due to Alzheimer’s disease, prodromal Alzheimer’s disease, diagnostic criteria, biomarker, memory clinics

## Abstract

**Objectives**: Mild cognitive impairment (MCI) is a heterogeneous clinical syndrome and is important for the diagnosis and management of Alzheimer’s disease (AD). With the expansion of biomarker-based diagnostics, the aim of this study is to clarify the current attitudes towards and the use of MCI, and MCI due to AD, in German memory clinics. **Methods**: An online survey (50 items) was performed in 2022 among specialized clinicians (*N* = 45) in German memory clinics to assess the use of MCI and biomarkers in current diagnosis and treatment. Attitudinal and frequency items were assessed with a five-point numeric scale (*strongly disagree* = 1 to *completely agree* = 5 and *never* = 1 to *always* = 5, respectively). **Results**: All respondents used MCI as a clinical diagnosis. The benefits of diagnosing MCI were labeling deficits as disease symptoms (*M* = 4.4, *SD* = 0.7), improving coping with symptoms (*M* = 4.1, *SD* = 0.9), and motivating risk reduction activities (*M =* 4.0, *SD* = 0.9). Overall, 37 respondents used specialized diagnostic criteria for MCI due to AD, and all had access to biomarker diagnostics. Patients with MCI due to AD received more frequent counseling on memory training (*p* < 0.001), other non-pharmacological treatments (*p* < 0.001), and antidementive drug treatment (*p* < 0.001) than patients with MCI of other etiologies. Acetylcholinesterase inhibitors were prescribed significantly more frequently to patients with MCI due to AD (*p* < 0.001) compared to other MCI patients. **Conclusions**: MCI is commonly used as a clinical diagnosis in German memory clinics. AD biomarker assessment is well established and influences patient counseling and treatment recommendations.

## 1. Introduction

Alzheimer’s disease (AD) is the most common cause of dementia and places a significant burden on patients, their families, caregivers, and societies. AD is a clinico-pathological continuum with symptoms spreading progressively from subjective cognitive complaints relating to mild cognitive symptoms to dementia over several decades [1]. Considering all stages, updated global estimates state that 416 million persons worldwide are on the AD continuum, the majority being in the early clinical stages of AD [2]. In Germany, the current dementia prevalence is 1.8 million people, with the majority classified as having Alzheimer’s dementia. This number is expected to increase to 2.7 million by 2050 [3]. No valid data exist for early clinical stages, i.e., mild cognitive impairment (MCI).

With the emergence of new AD treatment paradigms in the form of disease-modifying therapies (DMTs), the diagnostic focus is shifting to the MCI stage of AD with biomarker-confirmed diagnosis [4]. By using biomarkers, patients with MCI due to AD can be detected precisely among the heterogeneous etiologies of MCI in the elderly [5]. Since November 2023, biomarker-based diagnosis in early clinical AD stages has been added to the German S3 Dementia Guideline [6]. This aims at an early intervention for early AD patients, including social counseling and, potentially, based on the indication of DMTs.

However, clinical practice and diagnostic workflow—even in specialized memory clinics—cannot be considered fully standardized currently [7], delaying timely biomarker-based diagnosis in MCI patients. An understanding of clinicians’ access to diagnostic tools and the current use of diagnostic terms and definitions, along with related attitudes, is missing for MCI, particularly at national levels. The aim of this study is to clarify current attitudes, both towards the concept of MCI and the use of biomarkers in these patients, among German memory clinic specialists. An online survey was conducted to gather empirical information about existing clinical practices. The survey built upon previous research by Bertens et al. [8] and Roberts et al. [9] involving American and European specialists. Particular focus was placed on the differences in the assessment and management (counseling and treatment) of (1) MCI in general and (2) MCI due to AD.

## 2. Methods

### 2.1. Online Survey

A cross-sectional online survey was designed with 50 quantitative and qualitative items. It was adapted from surveys by Bertens et al., 2019 [8], and Roberts et al., 2010 [9]. Modifications included translation into German and alignment with European classifications [10]. Additional items covered drug prescription and attitudes towards new drugs. The survey comprised three parts:

1. Current terms, definitions, clinical practices, and the management of MCI, including new therapeutic strategies like DMTs, as well as attitudes towards MCI and MCI due to AD.
2. Paired questions on counseling topics and pharmacological treatment for both forms of MCI.
3. Socio-demographic and center-based characteristics.

Questions were mainly closed (nominal, ordinal, or interval-scaled, and single- and multiple-choice), with items rated on five-point scales (1 = *strongly disagree* to 5 = *completely agree*). Frequency items (e.g., diagnostic methods, counseling, drug prescription) were also assessed using five-point scales (1 = *never* to 5 = *routinely*). The survey was pre-tested for content and feasibility; in a pilot test, all co-authors individually completed the survey and gave written feedback to GSB, who amended the text accordingly. MB reframed items to allow for quantitative statistical analysis. The final text was formatted for online administration via LimeSurvey, hosted by the GWDG. The average completion time was *M* = 37.25 ± 27.43 min.

### 2.2. Study Procedures

Ethical approval was not required as the survey focused on professional/institutional practices without collecting individual patient data. A total of 58 memory clinics (MCs) were contacted, with 32 from the German Network of Memory Clinics (DNG) and 26 identified via public databases. A total of 30 MCs were located in psychiatric institutions, 26 in a neurologic institutions and 2 in a geriatric institution. A maximum of three participants (department head, employee with medical background, employee with non-medical background) were requested at each center, resulting in a total of 174 possible participants. The survey link was sent to department heads and forwarded to specialists. E-mail reminders were sent twice. Data collection occurred between October 2021 and December 2022.

### 2.3. Statistical Analyses

IBM SPSS^®^ Statistics software v29 was used for data analysis. For descriptive representation, frequencies (*n*, %) and means with standard deviations (*M* ± *SD*) were calculated. Differences between MCI due to AD vs. MCI of other etiologies in individual numerical ratings were tested for significance for two variable groups (primary outcomes), which contained paired questions for both MCI forms: (1) counseling topics (11 paired questions) and (2) pharmacological treatment (7 paired questions). In sum, we calculated 18 *t*-tests for paired samples with corresponding effect sizes (*d_emp_*). Because of α-error inflation, we adjusted the level for statistical significance using the Bonferroni method: starting from α = 0.05 (two-sided testing), the critical level was set at α_adj_ = 0.05/18 = 0.00278. Thus, *p*-values < 0.003 were considered significant. Additional explorative analyses were performed on two binary variables differentiating between MCI due to AD and MCI of other etiologies (single variable: “lumbar puncture for assessment of CSF biomarkers”; variable group: “explanation of MCI diagnosis towards patients and caregivers”). For these, multiple McNemar tests were calculated, with no α correction (significance was determined at *p* < 0.05; see results section for details). In further exploratory post hoc analyses examining management differences between neurologists and psychiatrists, ten two-sided Welch *t*-tests were performed to compare differences in the frequency of drug prescription, and sixteen two-sided Welch *t*-tests were calculated to assess differences in counseling frequency. Descriptive statistics were calculated for each variable prior to analysis. The Welch *t*-test was chosen due to differences in group sizes [11]. No α correction was made for these explorative analyses, with significance set at *p* < 0.05.

## 3. Results

### 3.1. Sample Characteristics

The survey was completed by 45 memory clinic specialists (response rate of 25.9%). Most of the specialists had a medical background (*n* = 37; 82.2%) (see Table 1).

### 3.2. Availability of Diagnostic Tools and Assessments in Participating Memory Clinics

All (*N* = 45) respondents stated that their clinics provided access to CSF diagnostics and routine bloodwork. The majority had access to detailed neuropsychological testing (*n* = 44; 97.8%), cranial MRI (*n* = 43; 95.6%), EEG (*n* = 40; 88.9%), cranial CT and FDG-PET (*n* = 37; 82.2% each), genetic diagnostics (*n* = 34; 75.6%), amyloid-PET (*n* = 33; 73.3%), and SPECT (*n* = 27; 60%). The minority (*n* = 17, 37.8%) had access to Tau-PET.

All *N* = 45 participants recognized MCI as a clinical diagnosis. The most commonly used term was MCI (*N* = 45, 100%), followed by cognitive impairment/no dementia (CIND) (*n* = 9, 20.0%). Age-associated memory impairment (AAMI) was not used. Less common terms for cognitive impairment were only used by single participants (see Table 2). The majority of participants (*n* = 41; 91.0%) differentiated between amnestic vs. non-amnestic MCI, and *n* = 27 (60.0%) used the additional subtypes of single domain vs. multiple domain MCI.

### 3.3. MCI

A majority of participants (*n* = 35, 77.8%) treated MCI patients “routinely” (>10 patients/month), followed by “sometimes” (*n* = 7, 15.6%; 6–10 patients/month) and “rarely” (*n* = 3, 6.7%; <5 patients/month). The most common description of cognitive deficits given to MCI patients and caregivers were “memory problems” (*n* = 39, 86.7%) and “mild cognitive impairment/disorder” (*n* = 35, 77.8%), with “brain dysfunction” being less common (*n* = 10, 22.2%).

The conditions were communicated as “possibility of early Alzheimer’s disease” (*n* = 25, 55.6%), “possible early dementia” (*n* = 18, 40.0%), and “possibility of a neurodegenerative process” (*n* = 17, 37.8%). A minority (*n* = 7, 15.6%) chose “exclusion of dementia or Alzheimer’s disease”.

The majority of participants included risk factors such as MCI subtype (*n* = 40, 88.9%), medical (*n* = 40, 88.9%) and psychiatric comorbidities (*n* = 39, 86.7%), family history (*n* = 39, 86.7%), and age (*n* = 37, 82.2%) in the diagnostic process. Gender was mentioned by a minority (*n* = 13, 28.9%). Additionally, “degree of cerebral microangiopathy”, “neurological comorbidities”, and “level of education” were each mentioned by one participant.

**Figure 1:**
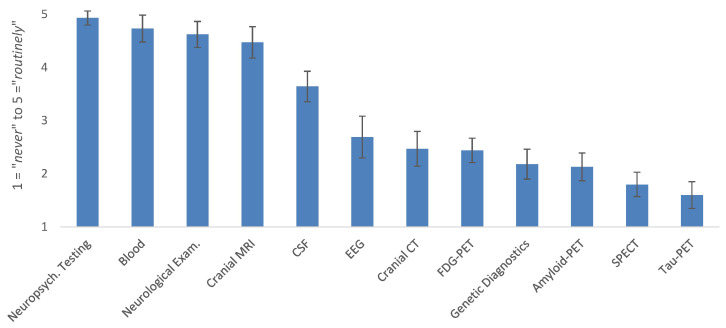
Frequency of diagnostic procedures in initial diagnostics in patients with MCI shown in bars with 95% CIs. Abbreviations: MRI, magnetic resonance imaging; CSF, cerebrospinal fluid; EEG, electroencephalography; CT, computer tomography; FDG-PET, fluorodeoxyglucose-positron emission tomography; SPECT, single-photon emission computed tomography.

In the initial diagnostic assessment of MCI patients, detailed neuropsychological testing (NPT) was used most frequently (4.9 ± 0.4; rated on the five-point numeric scale ranging from (1) *never* to (5) *routinely*), followed by laboratory blood tests (4.7 ± 0.9), neurological examination (4.6 ± 0.8), cranial MRI (4.5 ± 1.0), and CSF biomarkers for AD and neurodegeneration (3.6 ± 1.0; see Figure 1 for an overview). Functional imaging and genetic diagnostics were used less frequently (all ≤ 2.5) in initial assessments. On average, respondents reported spending 70.9 ± 43.1 min on initial assessments of MCI or dementia.

### 3.4. MCI Due to AD

In sum, *n* = 21 (46.7%) of specialists reported having knowledge about specific criteria for MCI due to AD, NIA-AA criteria for MCI due to AD [12], and IWG criteria for prodromal AD [13]. *n* = 21 (46.7%) were familiar only with NIA-AA criteria, and *n* = 3 (6.7%) only with the IWG criteria.

In clinical practice, most specialists (*n* = 37; 82.2%) used one of the following criteria: NIA-AA (*n* = 32; 86.5%), IWG (*n* = 11; 29.7%), or NINCDS/ADRDA [14] (*n* = 6; 16.2%). Reasons for the use of criteria were simplicity, anchoring in research, ease of use, best suitability for clinical practice, being based on biomarkers, and being the standard criteria in their institution or clinical practice (free responses).

The majority of specialists who used specific criteria for MCI due to AD also claimed to diagnose patients frequently and treat them routinely (>1 patient/month; *n* = 24; 64.9%); whereas *n* = 11 (29.7%) treated patients at least sometimes (monthly). In addition, *n* = 37 (51.8%) reported using specific MCI due to AD criteria in the clinical workup of all MCI patients to identify AD. All respondents who used criteria for MCI due to AD or prodromal AD stated that they always informed patients and family members of their diagnosis.

**Figure 2:**
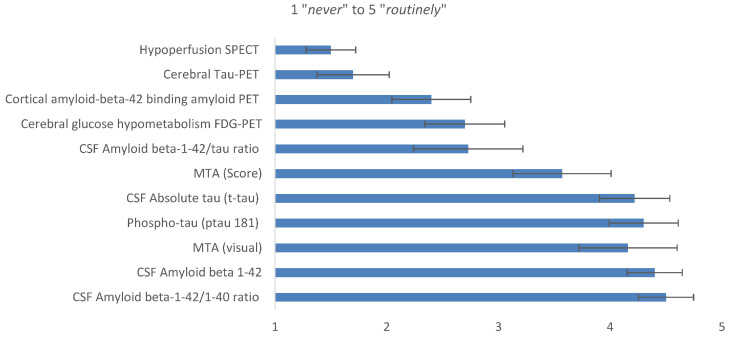
Frequency of biomarker use for diagnosis of MCI due to AD, rated on a five-point numeric scale (two wording anchors: 1 = *never* to 5 = *routinely*) by memory clinic specialists with 95% CIs (*n* = 37). Legend: SPECT, single-photon emission computed tomography; PET, positron emission tomography; FDG, fluorodeoxyglucose; MTA, medial temporal lobe atrophy; CSF, cerebrospinal fluid.

Participants rated commonly used biomarkers for diagnosis of MCI due to AD on a five-point numeric scale, ranging from (1) *never* to (5) *routinely*. CSF amyloid beta-1-42/1-40 ratio (Aβ-ratio) reached the highest rating (4.5 ± 0.8), followed by CSF amyloid beta 1-42 (Aβ1-42; 4.4 ± 0.8), CSF phospho-tau (ptau 181; 4.3 ± 1.0), CSF absolute tau (t-tau; 4.2 ± 1.0), and medial temporal lobe atrophy (MTA) determined by visual/qualitative MRI rating (4.2 ± 1.0) and MTA Score (3.6 ± 1.4). The use of other markers was rated with lower frequencies (see Figure 2).

### 3.5. MCI Due to AD vs. Other MCI

Results for attitudes towards MCI due to AD and other MCI are shown in Table 3. For both MCI due to AD and other MCI etiologies, specialists agreed that diagnosis supports patients and caregivers in symptom management, active future planning, treatment, and risk reduction. Specialists did not agree with the statement that diagnosis of MCI/MCI due to AD causes unnecessary worries for patients and family members, and that it cannot be diagnosed precisely.

**Figure 3:**
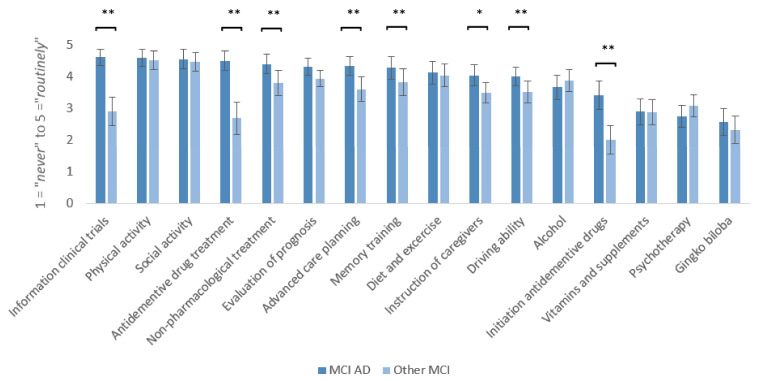
Frequency of counseling topics for MCI due to AD and MCI of other etiologies, rated on a five-point numeric scale (two wording anchors: 1 = *never* to 5 = *routinely*) by memory clinic specialists (*n* = 37) * *p* < 0.05, ** *p* < 0.001.

Ratings for the frequency of counseling topics were compared for MCI due to AD and MCI of other etiologies (see Figure 3). For therapeutic aspects, MCI due to AD patients were counseled significantly more frequently on information about clinical trials (*t*(36) = 8.31, *p* < 0.001, *d_emp_* = 1.37), antidementive drug treatment (*t*(36) = 6.90, *p* < 0.001, *d_emp_* = 1.14), initiation of antidementive drug treatment (*t*(36) = 6.67, *p* < 0.001, d_emp_ = 1.10, non-pharmacological treatment (e.g., occupational therapy; *t*(36) = 3.90, *p* < 0.001, d_emp_ = 0.64), and memory training (*t*(36) = 3.64, *p* < 0.001, d_emp_ = 0.60). For management strategies, advanced care planning (*t*(36) = 4.62, *p* < 0.001, d_emp_ = 0.76), instruction of caregivers (*t*(36) = 3.42, *p* = 0.002, *d_emp_* = 0.56) and driving ability (*t*(36) = 3.85, *p* < 0.001, d_emp_ = 0.63) reached significantly higher ratings in MCI due to AD. Evaluation of prognosis (*t*(36) = 2.90, *p* = 0.006, *d_emp_* = 0.48) missed significance due to Bonferroni correction (critical *p*-value at <0.003). For all remaining pairwise comparisons, no significant differences were found.

In the free response section, specialists supplemented further topics: counseling of family members, antidepressants, regular check-ups and monitoring, clinical studies, psychoeducation and in-house social counseling (*n* = 1 each).

**Figure 4:**
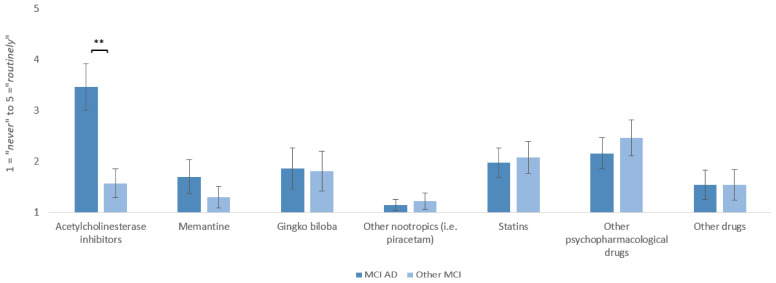
Frequency of medication prescription in MCI due to AD and other MCI rated on a five-point numeric scale (two wording anchors: 1 = *never* to 5 = *routinely*) by memory clinic specialists (*n* = 37) ** *p* < 0.001.

Specialists prescribed Acetylcholinesterase inhibitors (AChE-I) significantly more frequently to patients with MCI due to AD compared to patients with MCI of other etiologies (*t*(36) = 7.84, *p* < 0.001, *d_emp_* = 1.29). No significant differences in prescription frequency were found for other medications or supplements (see Figure 4). In a free response section, antidepressants were commonly mentioned as further medication use in MCI/MCI due to AD patients (*n* = 15).

Ratings of attitudes regarding new and future disease-modifying therapies are shown in Table 4. On average, specialists stated they would recommend, communicate and discuss AD biomarkers and prognosis with MCI patients if DMTs were available.

### 3.6. Explorative Analysis: Lumbar Puncture for Assessment of CSF Biomarkers and Explanation of MCI Diagnosis

For the explorative analysis, binary variables (“chosen” vs. “not chosen”) were analyzed. Lumbar puncture for assessment of CSF biomarkers was chosen more frequently as a decisive diagnostic measure in MCI due to AD (*n =* 43, 95.6%) compared to other MCI etiologies (*n = 35*, 77.8%). The difference reached significance (*p* = 0.021).

#### 3.6.1. Explorative Analysis: Explanation of Diagnosis

**Figure 5:**
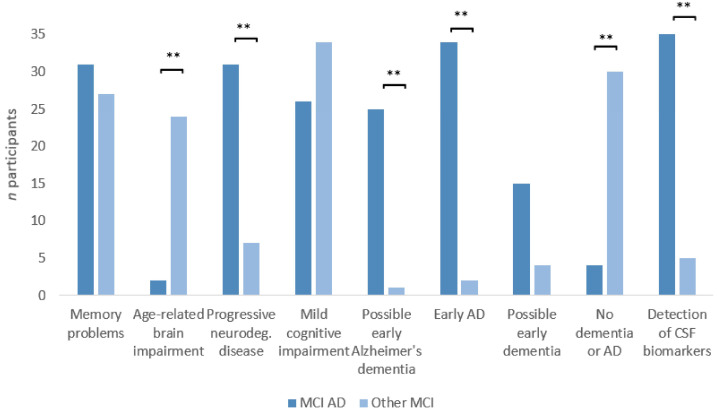
Commonly used terms to explain a given clinical diagnosis to patients and caregivers. Columns show the response frequency to nine topics of information/counseling for a diagnosis of MCI due to AD (beginning Alzheimer’s disease) vs. MCI of other etiologies. ** indicates that the frequency of response was significantly different between the two groups (*p* < 0.001).

The terms “progressive neurodegenerative disease”, “possible early Alzheimer’s dementia, “early Alzheimer’s disease”, and “Detection of CSF Biomarker” were used more frequently to explain a diagnosis of MCI due to AD, while “age-related brain impairment” and “no dementia or Alzheimer’s disease” were used more frequently to explain MCI of other etiologies. The term “Memory problems” was used for both conditions (see Figure 5).

#### 3.6.2. Explorative Analysis: Prescribing Medication and Counseling Differences Between Neurologists and Psychiatrists

There were no significant differences in the management of patients regarding prescription and counseling frequency between neurologists and psychiatrists, with one exception: psychiatrists prescribed “other psychopharmacological drugs” more often than neurologists for patients with MCI of other etiologies (*t*(21) = 2.18, *p* = 0.041).

## 4. Discussion

This survey aimed to assess the clinical use of the term MCI, as well as diagnostic and treatment procedures in specialized memory clinics in Germany in 2022. Our survey shows that MCI is firmly established as a clinical diagnosis (100% acceptance) and is recognized as a clinical syndrome of AD. Previous surveys showed lower acceptance rates among European specialists in 2019 (92%) [8] and American specialists in 2009 (90%) [9]. This reflects the growing recognition of MCI and developments in the field, in line with 2023 findings from the US, where surveyed specialists reported feeling confident in diagnosing MCI [15]. In this study, all specialists had access to biomarker assessments and used specific criteria (NIA-AA and IWG) [12,13] for diagnosing MCI due to AD or prodromal AD (82%) by biomarkers, a higher percentage than previously reported in 2019 (68% of European specialists) [8].

After neuropsychological assessment and exclusion of secondary causes, assessment of AD-biomarkers with cranial MRI and CSF (Aβ1-42 ratio, Aβ1-42, ptau 181, t-tau) were the most frequent diagnostic procedures. This reflects a homogeneous diagnostic pathway aligned with current national guidelines. Almost all specialists (95.6%) stated that CSF-biomarker assessment is crucial for diagnosing MCI due to AD, more so than for other MCI types (77.8%). The increased use of amyloid markers has likely resulted from more widespread awareness of their diagnostic value and full access to diagnostic procedures in university centers. Current German guidelines recommend the assessment of CSF biomarkers if a change in clinical management is expected [6].

Functional imaging such as PET was used the least, likely due to its limited availability and the lack of reimbursement for its use by public health insurance in Germany [7]. Despite AD biomarker assessment, respondents distinguished MCI neuropsychologically into amnestic and non-amnestic subtypes, with the amnestic subtype having a higher risk of progressing to Alzheimer’s dementia [16].

MCI due to AD was more frequently communicated to patients as a neurodegenerative disease, emphasizing its progressive nature and impact on individual prognosis. These patients were counseled on treatment strategies more often, both pharmacological and non-pharmacological, and informed about clinical trials. Specialists also initiated AChE-I treatment in patients with MCI due to AD as an off-label medication, a common practice noted in previous surveys [8,9]. Due to the lack of approved treatments for pre-dementia AD (as of summer 2024), clinicians may prescribe AChE-I, especially in patients with short-term memory deficits [17]. Our results indicated that psychiatrists tend to medicate MCI patients of other etiologies more comprehensively than neurologists. It must be kept in mind that there are two different concepts to describe this clinical population, the extended Alzheimer’s Association (AA) framework [18] and the International Working Group criteria [13], with different implications.

Counseling for patients with MCI due to AD more frequently included the topics symptom progression, loss of autonomy, and advanced care planning, and involves patients and families in early decision-making. Overall, respondents agreed that biomarker-based MCI diagnosis benefits patients and caregivers, aligning with findings from a 2023 meta-analysis [19]. Knowledge of positive AD biomarker status has been found to promote lifestyle changes with a preventive impact on disease progression [20]. From a therapeutic perspective, DMTs reducing cerebral amyloid pathology in early AD have been approved in the US [21] and several other countries [22]. The CHMP issued a recommendation for the approval of Lecanemab in the EU in November 2024 [23]. Hence, DMTs are expected to be available in Germany in the foreseeable future. Diagnostic assessment of AD biomarkers will be mandatory for treatment with anti-amyloid DMTs [24]. German specialists are aware of this development and are likely to recommend AD biomarker assessments and inform patients about Aβ1-42 status following the approval of new DMTs. Previous studies have shown a high interest in learning about AD biomarker status among patients, especially those with a family history of AD [25,26]. Furthermore, the field is progressing towards incorporating biomarkers into the diagnostic process for cognitive impairment, as demonstrated by the recently revised 2024 AA workgroup criteria for the diagnosis and staging of Alzheimer’s disease [27]. Biomarker assessment is thus becoming increasingly important in clinical practice.

### Limitations and Strengths

The primary limitation of our study is the small (*n* = 45) and selective sample, which may reduce the study’s external validity and limits its generalizability. First, our sample is confined to specialized memory clinics in Germany, with 95.6% of responses coming from academic hospitals that may share similar characteristics, possibly limiting the applicability of findings to other settings. Second, physicians were overrepresented (82.2%), likely due to the survey’s focus on medical diagnostic procedures. Third, the response rate was low at 25.9% (45 out of 174 invited specialists), and more strategies to motivate non-responders may have been needed. A potential motivation bias linked to knowledge may have favored responses with positive attitudes towards using the concepts. Due to anonymization, it was not possible to further describe characteristics of responders and non-responders. Additionally, the survey’s length may have limited responses. Lastly, self-reporting is an inherent limitation to survey-based research.

However, our study’s strength lies in the highly selected sample of experienced German specialists. Our survey assessed the current management of MCI patients in specialized German memory clinics, providing detailed insights into their workflows. Importantly, we contributed innovative data on AD biomarker use in MCI, relevant for emerging diagnostic and therapeutic approaches in AD. Our findings support the feasibility of MCI due to AD biomarker criteria in clinical practice, as reflected in the current S3 guidelines for dementia [6], and may aid in harmonizing MCI diagnostics and improving care in Germany.

## 5. Conclusions

Our survey showed that MCI is frequently used to diagnose a clinical syndrome of AD in German memory clinics. AD biomarker assessment according to specific criteria for MCI due to AD is well established in the diagnostic process. AD biomarker status impacts patient management with respect to counseling of patients and caregivers as well as treatment recommendations. German memory clinics offer all necessary diagnostic procedures for MCI, which is crucial as future DMTs for early AD will require the assessment of cerebral amyloid status.

## Figures and Tables

**Table 1:** Descriptive data of participants (*N* = 45).

Item	
Age (*M* ± SD)	41.2 ± 9.0
Gender *n* female/total (%)	17/45 (37.8%)
*n* male/total (%)	24/45 (53.3%)
*n* n/a total (%)	4/45 (8.9%)
Medical specialty, n (%)	
Psychiatry	17 (37.7%)
Neurology	12 (26.6%)
*Nervenarzt* (Psychiatry + Neurology)	8 (17.8%)
Neuropsychology	8 (17.8%)
Years of practice (*M* ± SD)	9.2 ± 7.6
Further qualifications, *n* (%)	
Geriatric psychiatry	10 (22.2%)
Geriatric medicine	7 (15.6%)
Place of employment, *n (%)*	
University/academic hospital	43 (95.6%)
Non-academic teaching hospital	2 (4.4%)

**Table 2:** Terms and Definitions.

Multiple Selection Items	*n* (%)
Mild Cognitive Impairment (MCI)	45 (100%)
Cognitive Impairment/No Dementia (CIND)	9 (20.0%)
Age-Associated Memory Impairment (AAMI)	0 (0.0%)
Subclassification of MCI	
Amnestic vs. non-amnestic MCI	41 (91.1%)
Single vs. multiple domain MCI	27 (60.0%)
Free Response Items	
Incipient Dementia Syndrome	1 (2.2%)
Mild Cognitive Disorder	1 (2.2%)

Notes. Memory clinic specialists; *N* = 45.

**Table 3:** Attitudes of memory clinic specialists towards MCI due to AD vs. other MCI.

Item	*MCI* Due to *AD*		Other *MCI*	
	*M* ± *SD*	95% *CI*	*M* ± *SD*	95% *CI*
1. Labeling MCI (due to AD) deficits as symptoms of a disease is helpful for patients and family members.	4.5 ± 0.6	[4.3, 4.7]	4.4 ± 0.7	[4.2, 4.6]
2. The diagnosis of MCI (due to AD) as a disease helps families to cope with deficits in everyday life.	4.3 ± 0.8	[4.1, 4.5]	4.1 ± 0.9	[3.8, 4.4]
3. MCI (due to AD) diagnosis can be helpful for motivating patients to engage in risk reduction activities.	4.2 ± 0.8	[4.0, 4.4]	4.0 ± 0.9	[3.7, 4.3]
4. MCI (due to AD) diagnosis can support patients in planning for the future.	4.4 ± 0.9	[4.1, 4.7]	4.0 ± 0.9	[3.7, 4.3]
5. MCI (due to AD) diagnosis supports the patients and caregivers in advanced care planning.	4.3 ± 0.8	[4.1, 4.5]	3.9 ± 1.0	[3.6, 4.2]
6. Specific medication can be of use for MCI (due to AD) patients.	3.8 ± 1.2	[3.4, 4.2]	3.6 ± 1.1	[3.3, 3.9]
7. MCI (due to AD) diagnosis helps younger patient’s career planning.	4.0 ± 0.9	[3.7, 4.3]	3.4 ± 1.1	[3.1, 3.7]
8. MCI (due to AD) diagnosis supports families in financial planning.	4.0 ± 1.0	[3.7, 4.3]	3.3 ± 1.0	[3.0, 3.6]
9. MCI (due to AD) is better described as early Alzheimer’s disease.	3.1 ± 1.2	[2.7, 3.5]	1.8 ± 1.0	[1.5, 2.1]
10. MCI (due to AD) diagnosis causes unnecessary worries for patients and caregivers.	2.2 ± 1.1	[1.9, 2.5]	1.9 ± 0.7	[1.7, 2.1]
11. MCI (due to AD) is too complex to be reliably diagnosed.	1.6 ± 0.9	[1.3, 1.9]	1.5 ± 0.9	[1.2, 1.8]
12. There is no approved treatment for MCI (due to AD), so it is not useful to diagnose it.	1.5 ± 1.0	[1.2, 1.8]	1.3 ± 0.8	[1.1, 1.5]

Notes: respondents were asked to rate agreement to the following statements using a five-point numeric scale with two wording anchors ranging from 1 = strongly disagree to 5 = completely agree. MCI, mild cognitive impairment. *n* = 45.

**Table 4:** Impact of disease modifying therapies on diagnostic management of MCI.

*Following Aducanumab Approval in Europe I Will Be More Likely to…*	*M ± SD*
…recommend AD-biomarker diagnosis in MCI patients	3.4 ± 1.7
…communicate amyloid-status to MCI patients	3.2 ± 1.8
…discuss prognosis with MCI patients	3.1 ± 1.7
*…discuss prevention measures with MCI patients*	*3.0 ± 1.7*

Notes. Respondents were asked to rate agreement to these statements using a five-point numeric scale with two wording anchors, ranging from 1 = strongly disagree to 5 = completely agree. *n* = 45.

## Data Availability

Anonymized data will be made available to the scientific community upon reasonable request.

## Abbreviations

AChE-I: Acetylcholinesterase inhibitors; AD: Alzheimer’s disease; CSF: Cerebrospinal fluid; CT: Computed tomography; DMT: Disease-modifying therapies; DNG: German network of memory clinics (Deutsches Netzwerk Gedächtnisambulanzen); EADC: European Alzheimer’s Disease Consortium; EEG: Electroencephalography; FDG-PET: Fludeoxyglucose positron emission tomography; IWG: International Working Group; MCI: Mild cognitive impairment; MRI: Magnetic resonance imaging; MTA: Medial temporal atrophy; AA: Alzheimer’s Association; PET: positron emission tomography; SPECT: Single-photon emission computed tomography.

## References

[1] Aisen P.S., Cummings J., Jack C.R., Morris J.C., Sperling R., Frölich L., Jones R.W., Dowsett S.A., Matthews B.R., Raskin J. (2017). On the path to 2025: Understanding the Alzheimer’s disease continuum. Alzheimer’s Res. Ther..

[2] Gustavsson A., Norton N., Fast T., Frölich L., Georges J., Holzapfel D., Kirabali T., Krolak-Salmon P., Rossini P.M., Ferretti M.T. (2023). Global estimates on the number of persons across the Alzheimer’s disease continuum. Alzheimer’s Dement..

[3] Deutsche Alzheimer Gesellschaft e.V. (2020). Die Häufigkeit von Demenzerkrankungen.

[4] Tahami Monfared A.A., Phan N.N., Pearson I., Mauskopf J., Cho M., Zhang Q., Hampel H. (2023). A Systematic Review of Clinical Practice Guidelines for Alzheimer’s Disease and Strategies for Future Advancements. Neurol. Ther..

[5] Jack C.R., Bennett D.A., Blennow K., Carrillo M.C., Dunn B., Haeberlein S.B., Holtzman D.M., Jagust W., Jessen F., Karlawish J. (2018). NIA-AA Research Framework: Toward a biological definition of Alzheimer’s disease. Alzheimer’s Dement..

[6] Dgn E.V., Dgppn E. (2023). S3-Leitlinie Demenzen, Version 4.0. https://register.awmf.org/de/leitlinien/detail/038-013.

[7] Hausner L., Frölich L., von Arnim C.A., Bohlken J., Dodel R., Otto M., Rapp M., Schulz J., Supprian T., Wollmer M.A. (2021). Memory clinics in Germany—Structural requirements and areas of responsibility. Nervenarzt.

[8] Bertens D., Vos S., Kehoe P., Wolf H., Nobili F., Mendonça A., van Rossum I., Hort J., Molinuevo J.L., Heneka M. (2019). Use of mild cognitive impairment and prodromal AD/MCI due to AD in clinical care: A European survey. Alzheimer’s Res. Ther..

[9] Roberts J.S., Karlawish J.H., Uhlmann W.R., Petersen R.C., Green R.C. (2010). Mild cognitive impairment in clinical care: A survey of American Academy of Neurology members. Neurology.

[10] Dilling H., Mombour W., Schmidt M.H. (2015). ICD-10—Internationale Klassifikation Psychischer Störungen.

[11] Derrick B., Toher D., White P. (2016). Why Welchs test is Type I error robust. Quant. Methods Psychol..

[12] Albert M.S., DeKosky S.T., Dickson D., Dubois B., Feldman H.H., Fox N.C., Gamst A., Holtzman D.M., Jagust W.J., Petersen R.C. (2011). The diagnosis of mild cognitive impairment due to Alzheimer’s disease: Recommendations from the National Institute on Aging and Alzheimer’s Association workgroup. Alzheimer’s Dement..

[13] Cummings J.L., Dubois B., Molinuevo J.L., Scheltens P. (2013). International Work Group Criteria for the Diagnosis of Alzheimer Disease. Med. Clin. N. Am..

[14] McKhann G., Drachman D., Folstein M., Katzman R., Price D., Stadlan E.M. (1984). Clinical diagnosis of Alzheimer’s disease. Neurology.

[15] Gopalakrishna G., Brunton S., Pruzin J., Alford S., Hamersky C., Sabharwal A. (2023). Understanding the role of psychiatrists in the diagnosis and management of mild cognitive impairment and mild Alzheimer’s disease dementia: A cross-sectional survey. BMC Psychiatry.

[16] Gauthier S., Reisberg B., Zaudig M., Petersen R.C., Ritchie K., Broich K., Belleville S., Brodaty H., Bennett D., Chertkow H. (2006). Mild cognitive impairment. Lancet.

[17] Stage E., Svaldi D., Sokolow S., Risacher S.L., Marosi K., Rotter J.I., Saykin A.J., Apostolova L.G., Alzheimer’s Disease Neuroimaging Initiative (2021). Prescribing cholinesterase inhibitors in mild cognitive impairment-Observations from the Alzheimer’s Disease Neuroimaging Initiative. Alzheimer’s Dement..

[18] Dubois B., Villain N., Schneider L., Fox N., Campbell N., Galasko D., Kivipelto M., Jessen F., Hanseeuw B., Boada M. (2024). Alzheimer Disease as a Clinical-Biological Construct-An International Working Group Recommendation. JAMA Neurol..

[19] van der Schaar J., Visser L.N., Ket J.C., Groot C., Pijnenburg Y.A., Scheltens P., Bredenoord A.L., van den Hoven M.A., van der Flier W.M. (2023). Impact of sharing Alzheimer’s disease biomarkers with individuals without dementia: A systematic review and meta-analysis of empirical data. Alzheimer’s Dement..

[20] Kivipelto M., Solomon A., Ahtiluoto S., Ngandu T., Lehtisalo J., Antikainen R., Bäckman L., Hänninen T., Jula A., Laatikainen T. (2013). The Finnish Geriatric Intervention Study to Prevent Cognitive Impairment and Disability (FINGER): Study design and progress. Alzheimer’s Dement..

[21] FDA (2023). FDA Grants Accelerated Approval for Alzheimer’s Drug.

[22] EISAI US Eisai Receives Positive Opinion from the CHMP in the European Union for Lecanemab in Early Alzheimer’s Disease. https://media-us.eisai.com/2024-11-14-Eisai-Receives-Positive-Opinion-from-the-CHMP-in-the-European-Union-for-Lecanemab-in-Early-Alzheimers-Disease.

[23] Verband Forschender Arzneimittelhersteller e.V. Neue Alzheimer-Medikamente in Fortgeschrittener Entwicklung. https://www.vfa.de/de/arzneimittel-forschung/woran-wir-forschen/neue-alzheimer-medikamente-in-entwicklung.html.

[24] Pleen J., Camerucci E., Al-Sabbagh M., Cunningham K. (2024). Blood-Based Biomarkers in Alzheimer Disease: Clinical Implementation and Limitations. Pract. Neurol..

[25] Milne R., Diaz A., Badger S., Bunnik E., Fauria K., Wells K. (2018). At, with and beyond risk: Expectations of living with the possibility of future dementia. Sociol. Health Illn..

[26] Milne R., Bunnik E., Diaz A., Richard E., Badger S., Gove D., Jeanc G., Karinee F., Jose-Luise M., Katief W. (2018). Perspectives on Communicating Biomarker-Based Assessments of Alzheimer’s Disease to Cognitively Healthy Individuals. J. Alzheimer’s Dis..

[27] Jack R., Andrews J.S., Beach T.G., Buracchio T., Dunn B., Graf A., Ho C., Jagust W., McDade E., Molinuevo J.L. (2024). Revised criteria for diagnosis and staging of Alzheimer’s disease: Alzheimer’s Association Workgroup. Alzheimer’s Dement..

